# Visual Rehabilitation for Kindergarten Children with Developmental Delay: Case Series

**DOI:** 10.3390/children13050619

**Published:** 2026-04-29

**Authors:** Min-Muh Sheu, Hsi-Pao Hsieh, Chao-An Chi, You-De Shen, Ching-Ying Cheng

**Affiliations:** 1 Eye Health Promotion Centre of Eastern Taiwan, Hualien 970050, Taiwan; hsuhung@mch.org.tw; 2 Department of Ophthalmology, Mennonite Christian Hospital, Hualien 970050, Taiwan; cacabao@gmail.com; 3 Department of Special Education, National Taiwan Normal University, Taipei 106308, Taiwan; t14019@ntnu.edu.tw; 4 Department of Ophthalmology, Chung Shan Medical University Hospital, Taichung 402306, Taiwan; gogo95047@gmail.com; 5 Department of Optometry, Chung Shan Medical University, Taichung 402306, Taiwan

**Keywords:** developmental delay, visual rehabilitation, visual function, amblyopia

## Abstract

**Highlights:**

**What are the main findings?**

- Beyond standard optical correction, active visual rehabilitation can play a key role in overcoming sensory suppression, helping to establish stereopsis and stable oculomotor control.
- Improvements in visual efficiency were accompanied by positive educational and motor outcomes, such as faster reading speeds, fewer compensatory head movements, and better postural stability.

**What are the implications of the main findings?**

- The observed relationship between visual and gross motor development supports Dynamic Systems Theory, suggesting functional vision is actively constructed through sensorimotor exploration.
- Vision rehabilitation goals should be integrated into Individualized Education Programs (IEPs), relying on an interdisciplinary approach and strong family involvement for long-term success.

**Abstract:**

Purpose: This study investigated the effects of visual rehabilitation on binocular visual function in kindergarten children with developmental delay. Methods: This study comprised a case series tracking changes in binocular visual function in three children with developmental delay during rehabilitation. The rehabilitation schedule was tailored to the specific circumstances of each child and was divided into three phases—baseline, intervention, and maintenance—aiming to observe the impact of rehabilitation on visual performance. Results: All three children presented with pre-existing visual deficits—including amblyopia, strabismus, and oculomotor dysfunction—which were associated with functional challenges such as frequent falls, postural instability, and reading difficulties. Following the multidisciplinary rehabilitation program, marked improvements were observed in visual acuity, accommodative facility, and stereopsis. Furthermore, parents reported a decrease in daily functional challenges, with these gains being sustained during the post-intervention follow-up. Conclusions: This case series suggests that structured visual rehabilitation may improve binocular function and daily performance in children with developmental delays. These findings underscore that visual ability extends beyond mere visual acuity, highlighting the necessity of assessing multidimensional visual functions in special education and rehabilitative practice.

## 1. Introduction

In the early stages of child development, early detection and intervention are widely recognized as critical components in the implementation of special education. Treating developmental disorders or delays during the “critical period” (from birth to age six) yields the most profound functional plasticity [1]. Vision is integral to overall development, yet children with physical or cognitive disabilities often encounter significant systemic barriers to accurate diagnosis and timely treatment in general ophthalmology clinics [2,3]. Recent large-scale clinical evaluations have documented a staggeringly high prevalence of ocular morbidities in this population, ranging from significant refractive errors and strabismus to optic nerve abnormalities [4,5]. These systemic barriers frequently result in missed therapeutic opportunities, potentially impeding expected learning outcomes and long-term developmental milestones [6,7,8]. Furthermore, longitudinal insights suggest that even transient early visual deprivation can severely disrupt the maturation of neural circuits governing social–perceptual tasks [9].

Historically, early-intervention programs have lacked integrated collaboration among ophthalmologists, optometrists, occupational therapists, and special education teachers, thereby risking the clinical oversight of binocular integration issues. Binocular vision, predicated on the precise integration of bilateral sensory input, is the fundamental cornerstone of visual efficiency and the subsequent development of visual–motor coordination and literacy skills [10,11,12]. To address these diagnostic challenges, particularly in atypical development, current empirical evidence advocates for dynamic assessments like continuous tracking, which offer superior sensitivity compared to static clinical tests [13].

Crucially, visual functions critical for learning comprise two domains: visual efficiency and visual information processing. Deficits in visual efficiency frequently induce asthenopia and impair visually demanding tasks [14,15]. When visual efficiency is compromised, a “cognitive bottleneck” is established, forcing excessive neural resources toward foundational compensation and exacerbating learning deficits [16]. From a neurobiological perspective, these challenges are often framed within the “dorsal stream vulnerability” hypothesis, where neural pathways for spatial integration are uniquely susceptible to developmental insults [17,18]. Consequently, children with developmental delays often harbor “hidden” visual pathologies—such as impaired complex scene analysis—that remain undetected by conventional high-contrast optotype screenings [19].

The clinical significance of these conditions is underscored by their high prevalence, with ocular alignment and accommodative abnormalities affecting up to 33% and 61.7% of children, respectively [20,21]. Beyond localized eye strain, these abnormalities can trigger multifaceted systemic complications, including sleep disorders [22,23], motor skill deficits [24], and a loss of spatial awareness [25,26]. This interplay is particularly salient in neurobehavioral disorders, such as Attention-Deficit/Hyperactivity Disorder (ADHD), where the prevalence of binocular dysfunction is reportedly threefold higher than in the general population [27]. Common anomalies—including convergence insufficiency and poor fixation—markedly impair task accuracy and response times in these cohorts [28,29,30]. Notably, the symptomatic overlap between attention deficits and accommodative insufficiency often complicates diagnosis [31,32], while pharmacological treatments for ADHD may paradoxically exacerbate these visual symptoms, leading to blurred vision [33,34]. This complex interaction underscores the critical need for non-pharmacological interventions like vision therapy.

Furthermore, binocular vision underpins visual–motor integration; that is, the essential coordination of perception and motor control. Untreated problems manifest as difficulties in daily activities, such as fundamental self-care tasks or classroom writing [35,36]. Since accommodative facility and stereopsis are significant predictors of later reading ability [37], timely multidimensional intervention is vital to forestall further functional decline.

Vision rehabilitation (also known as vision training or therapy) is widely used to treat these dysfunctions, with recent evidence supporting immersive virtual reality as a transformative therapeutic tool for enhancing neural plasticity and binocular engagement [38,39,40]. Contemporary systematic reviews further highlight that targeted vision training can significantly optimize the coordination between visual and motor systems, which is essential for reaching developmental milestones in children [36,41]. While globally established as a multidisciplinary intervention for children with developmental challenges and literacy difficulties [42,43], vision rehabilitation remains largely underexplored in Taiwan, where clinical focus is primarily limited to conventional amblyopia management. This study investigated interdisciplinary approaches to address these visual and perceptual challenges. By integrating insights across disciplines, we sought to develop strategies that enhance visual efficiency and information processing, thereby informing evidence-based interventions aligned with the latest clinical findings [4,5].

## 2. Materials and Methods

### 2.1. Study Design and Ethical Considerations

This case study was approved by the Institutional Review Board of Human Experimentation Committee of Chung Shan Medical University Affiliated Hospital (CS1-24215). The research was conducted at a specialized optometry clinic by investigators certified in Good Clinical Practice (GCP). All procedures strictly adhered to the ethical tenets of the Declaration of Helsinki. Written informed consent was obtained from the parents or legal guardians of all participants prior to study enrollment.

### 2.2. Participants

Three children with developmental delays or a history of neurological insult were prospectively recruited from a kindergarten vision screening program. Inclusion criteria required the absence of ophthalmic disease and sufficient cognitive capability to cooperate with extended training protocols. The backgrounds are summarized as Table 1.

### 2.3. Clinical Assessments

A comprehensive standardized ophthalmic examination was performed at baseline. With the exception of the initial screening and objective refraction, all visual function evaluations were conducted under the participants’ best-corrected visual acuity (BCVA).

Anterior segment health was evaluated using slit-lamp biomicroscopy (SL-7E/7F; Topcon, Tokyo, Japan). Refractive status was objectively measured with an automated refractometer (KR-8100P; Topcon) and refined via static retinoscopy. Distance visual acuity was assessed using a View-M Digital Visual Acuity Chart. Binocular and accommodative functions were comprehensively profiled. Accommodative–vergence interactions and near-visual system coordination were evaluated using the Saladin Near Point Balance Card™ (Bernell Corp., Mishawaka, IN, USA). Horizontal and vertical deviations at distance and near were quantified utilizing either the prism cover test (PCT) or the Krimsky method, dictated by patient compliance. Stereopsis was measured via the Titmus Stereo Test (Stereo Optical Co., Chicago, IL, USA) to assess binocular fusion. Furthermore, the near point of convergence (NPC) and near point of accommodation (NPA) were determined using a Royal Air Force (RAF) ruler (Clement Clarke International, Harlow, Essex, UK).

### 2.4. Visual Rehabilitation Intervention

Individualized visual rehabilitation programs were formulated based on baseline clinical findings and implemented over a 6-month period. The treatment regimen comprised supervised, office-based therapy administered by a licensed optometrist—consisting of two 45 min sessions per week for 12 weeks—supplemented by a mandatory 20 min daily home reinforcement protocol. Daily occlusion therapy (patching for 2 h/day) served as a core component. Compliance was monitored via daily logbooks maintained by parents and reviewed weekly by the clinical team.

In addition to patching, binasal occlusion was integrated to manage esodeviations and mitigate over-convergence during binocular tasks [44]. To address the fundamental physiological link between binocular alignment and postural stability, vestibular–visual integration was facilitated using a balance board [45]. Specific orthoptic training modalities included: Accommodative Facility: Trained using ±2.00 D flipper lenses with 0.5 cm alphanumeric targets [46]. Fusional Vergence Facility: Trained utilizing 4Δ base-out and 4Δ base-in prisms [47]. Convergence and Spatial Awareness: A 6-foot Brock String was employed to stabilize convergence, while peripheral awareness cards were utilized to expand spatial attention [43]. Dynamic Ocular Tracking: Stimulated via a Marsden ball. Performance Monitoring: A computer-based vision therapy system (Bernell Corp.) provided real-time performance tracking and outcome evaluation [48]. Individualize rehabilitation methods for each case is summarized in Table 2.

## 3. Results

### 3.1. Interdisciplinary Approach and Intervention Protocol

**Figure 1:**
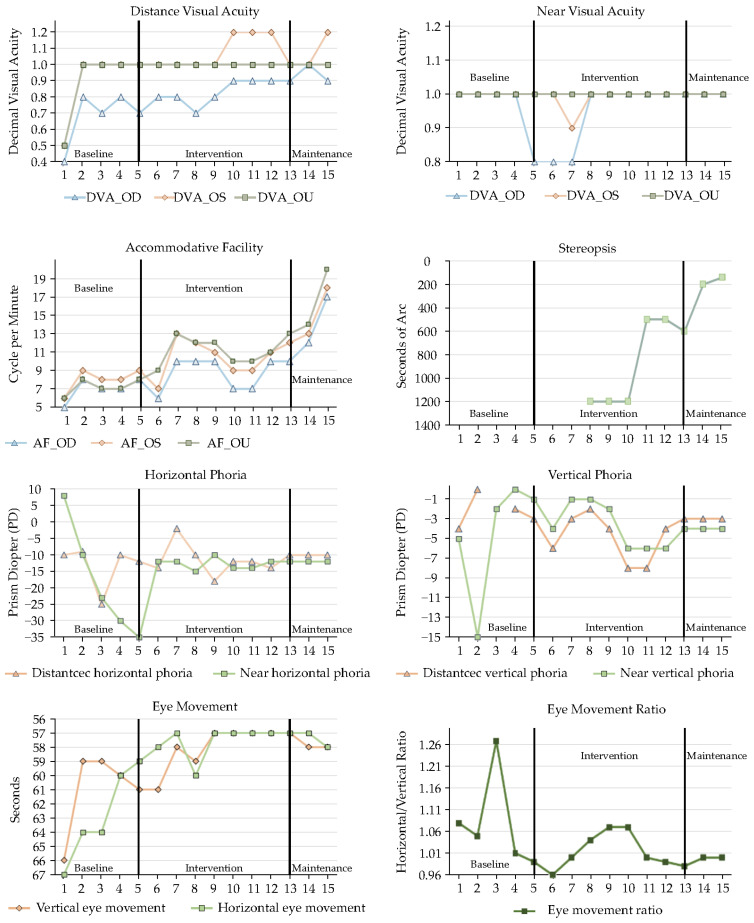
Visual function progression (Case 1).

**Figure 2:**
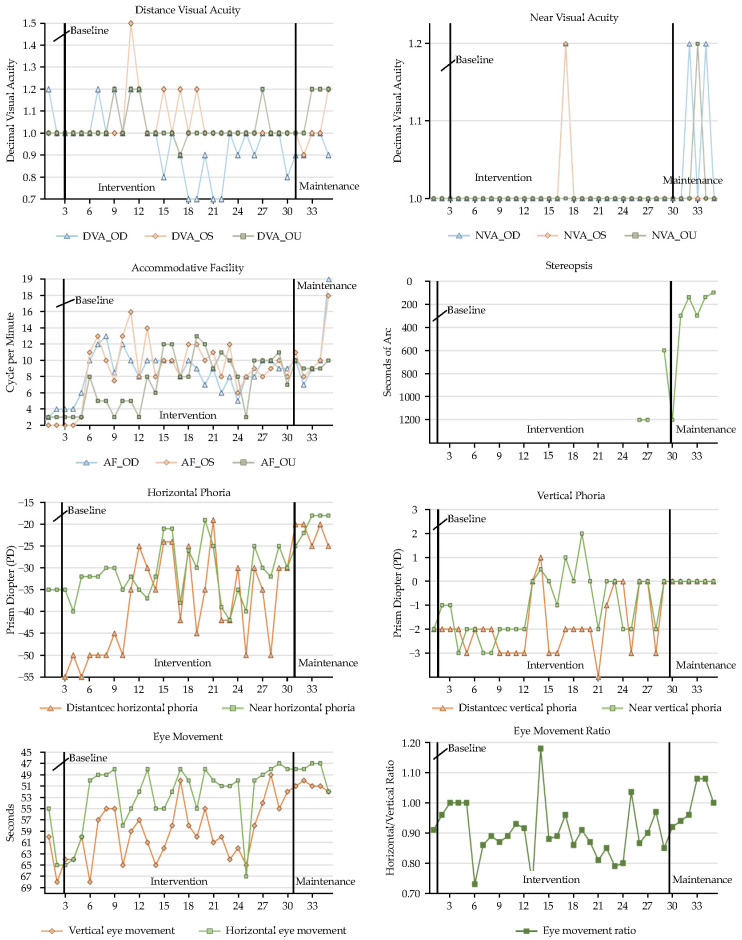
Visual function progression (Case 2).

**Figure 3:**
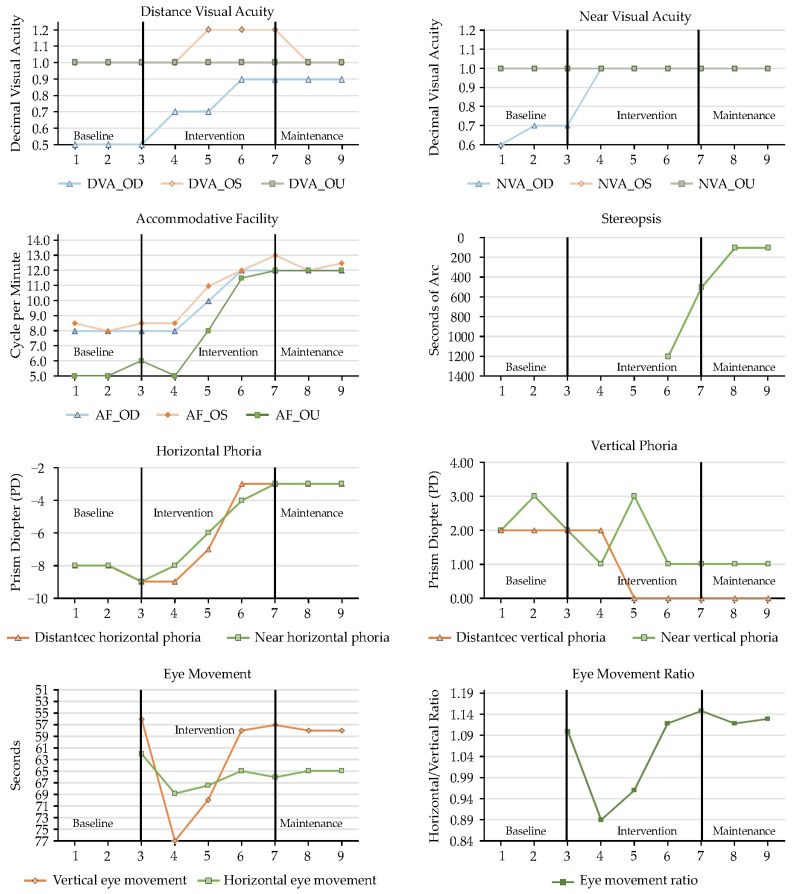
Visual function progression (Case 3).

This present study aimed to identify and manage young children with binocular vision dysfunction through a targeted kindergarten screening program. Following diagnosis, individualized visual rehabilitation programs were developed collaboratively, integrating clinical and pedagogical insights from optometrists, special education teachers, and occupational therapists. To rigorously distinguish the effects of active visual rehabilitation from simple optical correction, a structured intervention timeline was employed for the three enrolled children with developmental delays: an initial refractive adaptation phase (baseline), an active individualized visual rehabilitation phase (intervention), and a subsequent maintenance phase. A comprehensive comparison of baseline and post-intervention visual function parameters for all cases is provided in Table 3 and Figure 1, Figure 2 and Figure 3.

### 3.2. Visual Acuity and the Emergence of Stereopsis

While optimal optical correction effectively managed the refractive components of amblyopia by providing a clear retinal image, our findings underscore that the development of functional binocularity necessitates active, sensory–motor rehabilitative input to overcome deep-seated cortical suppression. In Case 1 (Figure 1), following the initial refractive adaptation period, distance visual acuity stabilized at 0.9–1.0 in the amblyopic eye. However, binocular integration was only achieved through structured orthoptic training. By the eighth training session, as interocular suppression was systematically reduced, primary stereopsis (140 arcseconds) successfully emerged, establishing a vital neurological foundation for higher-level binocular tasks.

The temporal dissociation between structural optical correction and sensory binocular fusion was most pronounced in Case 2 (Figure 2). Although his distance acuity normalized (1.0 bilaterally) during the initial adaptation period, stereopsis remained entirely absent, indicating a persistent sensory adaptation anomaly. It was only during the advanced intervention phase (Session 20+)—specifically following the introduction of the Brock string and targeted anti-suppression tasks designed to force simultaneous binocular perception—that this neurological barrier was breached. Consequently, stereopsis finally emerged, ultimately refining to a functional 100 arcseconds.

Similarly, Case 3 (Figure 3) demonstrated the clinical superiority of an active binocular approach over traditional passive penalization methods. Despite a documented history of unsuccessful occlusion therapy (patching 2 h/day)—which inherently fails to address the underlying interocular inhibition—the integration of active monocular training with progressive binocular demands yielded profound results. This active sensory stimulation not only improved his amblyopic eye distance acuity from 0.5 to 0.9 but also facilitated the de novo emergence of stereopsis (100 arcseconds) by the maintenance phase, a clinical milestone that remained unattainable through previous passive treatments.

### 3.3. Accommodative Facility and Binocular Alignment

The individualized interventions effectively enhanced accommodative flexibility and endurance, directly addressing the underlying ciliary muscle fatigue and accommodative dysfunction observed across all cases. Case 1 (Figure 1) demonstrated a remarkable recovery from subnormal accommodative facility, improving to over 15 cycles per minute (cpm) monocularly and reaching an optimal 20 cpm binocularly. This functional enhancement translated clinically to effortless focal shifts during near-viewing tasks. Case 2 (Figure 2), who initially presented with severely limited baseline facility (2–3 cpm), achieved a marked physiological shift to 10–18 cpm. This magnitude of improvement reflects true neuromuscular plasticity and enhanced accommodative stamina that surpassed potential test–retest artifacts. To rigorously rule out learning effects, varied alphanumeric targets and increased cognitive demands were systematically utilized during periodic assessments. Case 3 (Figure 3) also progressed from borderline baseline values to a highly stable 12 cpm, ensuring adequate accommodative reserves to sustain prolonged educational demands without asthenopia.

Regarding binocular alignment, the extreme baseline fluctuations characteristic of their developmental binocular instability was significantly mitigated. Case 1 (Figure 1) initially suffered from a severe, decompensating near exodeviation (peaking at approximately 35Δ) coupled with a substantial co-existing hypotropia (15Δ), which critically disrupted sensory fusion. Through targeted intervention, these volatile deviations were progressively consolidated into a stable, manageable 10Δ–12Δ exophoria and a minimal 3Δ–4Δ vertical deviation, bringing her alignment safely within her compensatory fusional reserves. For Case 2 (Figure 2), the large-angle esotropia gradually decreased in both magnitude and variability during active vergence training. Crucially, it was only after a foundational level of fusional vergence amplitude was actively established that single-vision glasses incorporating a 10Δ base-out therapeutic prism were successfully prescribed at Session 31. This sequential clinical approach—active training followed by prismatic support—effectively anchored his sensory fusion, leading to substantially decreased and stabilized deviations (distance: 25Δ, near: 18Δ). Finally, Case 3’s (Figure 3) initial presentation of an 8Δ esotropia and a mild but functionally significant hypotropia was practically resolved, yielding a highly stable 3Δ esophoria and near orthophoria. This robust postural alignment provides an optimal biomechanical foundation for dynamic visual processing and accurate depth judgment.

### 3.4. Oculomotor Control and Educational/Functional Outcomes

Crucially, the clinical improvements in visual system efficiency translated directly into significant gross motor and educational gains, underscoring the value of the interdisciplinary approach. Enhancements in visuo-motor integration were quantified using the Developmental Eye Movement (DEM) test. Case 1 (Figure 1) demonstrated a reduction in reading times (<60 s) with the complete elimination of compensatory head movements during saccades. For Case 2 (Figure 2), the strategic integration of home-based gross motor activities (balance board and trampoline) provided a stable vestibular–visual foundation, improving his DEM reading speed to 50 s and ameliorating his history of unsteady posture. Case 3 (Figure 3) exhibited dramatic oculomotor recovery, progressing from failing the entire horizontal DEM test (all 80 words misread) to normalized speed and accuracy (horizontal: 65 s, vertical: 60 s).

Beyond clinical metrics, caregivers and educational facilitators consistently reported remarkable functional transformations. These included significant reductions in falls and better stair negotiation (addressing core developmental delay symptoms), sustained reading interest, reduced crowding effects, and enhanced comprehension of mathematical word problems. These holistic outcomes confirm that the visual rehabilitation program not only normalized physiological ocular metrics, but also significantly bolstered the children’s overarching developmental and educational trajectories.

## 4. Discussion and Conclusions

Achieving best-corrected visual acuity (BCVA) is a fundamental prerequisite for visual development; however, attaining precise refraction in children with developmental delays is frequently hindered by cognitive or expressive limitations, which may lead to clinical misjudgments of refractive errors [4]. To rigorously address the concern that observed improvements might stem solely from optical correction, our study implemented a 6-week refractive adaptation period following the prescription of new optimal glasses. This protocol allowed us to clearly distinguish refractive adaptation—the passive resolution of optical blur—from the active functional gains achieved through structured visual rehabilitation. Interpreting our specific case results, the temporal dissociation observed—where stereopsis and stable oculomotor control emerged only after active vergence and anti-suppression training, well beyond the refractive adaptation phase—provides compelling clinical evidence. It demonstrates that while updated prescriptions provide a clearer retinal image, visual acuity alone is a limited metric that fails to capture the complexity of binocular stability, ocular motility, and higher-order visual perception [49,50].

From a neurophysiological perspective, optical correction merely addresses the “hardware” of the eye, whereas structured rehabilitation is strictly required to target the “software”—the cortical integration of visual signals [36,51]. Research suggests that for children with amblyopia and binocular dysfunction, passive patching or glasses may plateau; however, the addition of active sensory–motor stimulation (e.g., balance boards for vestibular–visual integration or binasal occlusion) can facilitate binocular summation and drive the emergence of stereopsis even long after refractive adaptation has stabilized [52,53].

Theoretically, these findings contribute significantly to the developmental literature, particularly aligning with Dynamic Systems Theory and embodied cognition paradigms [54]. Classical developmental theories often treated sensory, motor, and cognitive milestones as relatively independent tracks. However, our results challenge this modular view. The observation that interventions targeting vestibular–visual integration (e.g., Case 2) directly resolved both severe postural instability and oculomotor deficits suggests that visual development is inextricably linked to gross motor mastery. This adds to the recent developmental literature by reinforcing that functional vision is dynamically constructed through an active, sensorimotor exploration of the environment, rather than being a passive sensory input [55].

Given the complex profiles of these children, a multidimensional and ecological assessment is essential within the context of special education. This approach transcends traditional clinical metrics by integrating standardized functional vision evaluations with nuanced behavioral observations (e.g., compensatory head tilting or avoidance of near tasks) and comprehensive parental interviews. Such synergy allows for the identification of “functional vision” deficits that might be masked or overlooked in a standard exam. Practically, our findings suggest that practitioners should actively embed these visual-motor goals into the Individualized Education Program (IEP) framework. Furthermore, the design of our program aligns seamlessly with this IEP framework, transforming a strictly clinical intervention into a collaborative educational endeavor. The success of such a paradigm relies on a robust interdisciplinary support network comprising ophthalmologists, optometrists, special education teachers, and therapists. Most importantly, since much of the neuro-rehabilitative process occurs through repetitive at-home exercises, family-centered care and parental adherence emerge as the primary determinants of long-term success [56,57].

However, a critical methodological limitation of this case series must be acknowledged: the absence of a control group. It is well-recognized that developmentally delayed children may naturally perform better on vision screening and outcome tests over time, particularly when provided with continuous encouragement and practice. Consequently, without a matched control group, we cannot definitively isolate the specific therapeutic effects of our structured interventions—such as the 2 h of daily patching—from natural maturation, learning effects, or the general benefits of increased attention. Therefore, the outcomes reported herein should be interpreted as preliminary observations rather than definitive causal conclusions. Based on these limitations and the interpretations of the present study, future research should transition from case-based evidence to larger-scale, randomized controlled trials (RCTs) with appropriate control groups to rigorously quantify the efficacy of specific rehabilitation modalities across various developmental disorders. Furthermore, future program development should prioritize establishing standardized, easily accessible visual screening protocols and home-based therapy toolkits tailored specifically for early intervention centers [58]. Ultimately, the translation of these physiological visual gains into improved psychological well-being, gross motor confidence, and academic engagement validates the profound potential of this comprehensive, individualized, and interdisciplinary approach.

## Figures and Tables

**Table 1:** Baseline Clinical Characteristics and Visual Profiles of the Study Participants.

Case Age Gender	Non-Cycloplegic Refraction and Spectacle Prescriptions	Medical History and Ocular Diagnosis	Best-Corrected Distance Visual Acuity (BCVA)	Binocular and Stereoscopic Vision	Functional Vision
Case 1 5 Female	OD: +4.25 − 1.75 × 180 OS: +3.00 − 1.75 × 010	Developmental delay, hyperopia, astigmatism, bilateral amblyopia, right eye esotropia.	OD: 0.4 (20/50) OS: 0.5 (20/40) Near VA at 1.0 bilaterally	Absent stereopsis.	Parents reported prominent dynamic balance deficits, characterized by frequent falls, particularly when navigating stairs
Case 2 5 Male	OD: +2.00 − 2.00 × 175 OS: +4.00 − 4.00 × 180	Acquired brain injury at 18 months, congenital hyperopia, high astigmatism, left eye esotropia with amblyopia. (Previous bifocal prism spectacles yielded no improvement.)	Bilaterally: 1.0 (20/20) Normalized after a 6-week adaptation period, ruling out permanent amblyopia	Profound binocular dysfunction, alternating monocular suppression, absent stereopsis.	Significant postural instability
Case 3 5.5 Male	OD: +5.50 − 1.50 × 150 OS: +1.00 − 0.50 × 090	Developmental delay, hyperopia, astigmatism.	Distance: OD: 0.5 (20/40), OS:1.0 (20/20) Near: OD: 0.6 (20/40), OS: 1.0 (20/20) (Recorded after a 6-week adaptation period)	Severe depth perception deficits.	Poor visuo-motor integration, manifesting as frequent falls on stairs and marked apprehension toward dynamic visual tracking tasks, such as ball games

**Table 2:** Summary of Individualized Visual Rehabilitation Strategies.

Case	Core Rehabilitation Focus	Specific Training Methods and Strategies
Case 1	Structured Orthoptics and Anti-Suppression	1. Systematic Orthoptic Training: Step-by-step reduction of interocular suppression. 2. Accommodative Facility Enhancement: Intensive training using ±2.50 D flipper lenses. 3. Fusional Reserve Training: Targeted intervention for severe near exodeviation and hypotropia.
Case 2	Anti-Suppression Tasks, Sequential Prismatic Support and Vestibular–Visual Integration	1. Forced Binocular Perception: Integration of a Brock string and targeted anti-suppression tasks. 2. Sequential Approach: Active vergence training to build a foundation, followed by prescribing 10Δ base-out therapeutic prisms at Session 31. 3. Gross Motor Integration: Home-based activities utilizing a balance board and trampoline.
Case 3	Active Monocular Stimulation and Progressive Binocular Demands	1. Active Monocular Training: Replaced unsuccessful passive occlusion (patching) with active sensory stimulation. 2. Progressive Binocular Demands: Gradually increasing binocular coordination requirements built upon the active monocular foundation. 3. Dynamic Spatial–Visual Integration: Reconstructing central and peripheral vision through multi-directional gross motor tasks

**Table 3:** Comparison of baseline and post-intervention visual function parameters.

Visual Parameter	Assessment Phase	Case 1	Case 2	Case 3
Distance Visual Acuity (BCVA)	Baseline	OD: 0.4, OS: 0.5 OD: 0.7–0.8 *, OS: 1.0 *	1.0 OU *	OD: 0.5 *, OS: 1.0 *
	Post-Intervention	OD: 0.9–1.0, OS: 1.0–1.2	1.0–1.2 OU	OD: 0.9, OS: 1.0
Near Visual Acuity	Baseline	1.0 OU	1.0 OU	OD: 0.6, OS: 1.0
	Post-Intervention	1.0 OU	1.0 OU	OD: 1.0, OS: 1.0, 1.0 OU
Stereoacuity	Baseline	Absent	Absent	Absent
	Post-Intervention	140–200 arcsec	100–200 arcsec	100 arcsec
Accommodative Facility (cpm)	Baseline	OD: 5–6, OS: 7–9, OU: 6–8	OD: 4, OS: 2, OU: 3	OD: 8–9, OS: 8–9, OU: 5–6
	Post-Intervention	OD OS: 11–15, OU: 13–19	OD OS: 10–18, OU: 8–10	OD: 12, OS: 12, OU: 12
Horizontal Phoria	Baseline	Dist: 10Δ Eso (unstable): Near: 8Δ Exo (unstable):	Dist & Near: Large-angle Eso	Dist & Near: 8Δ Eso
	Post-Intervention	Dist & Near (stable): 10Δ–12Δ Eso	Dist: 25Δ Eso Near: 18Δ Eso **	Dist & Near: 3Δ Eso
Vertical Phoria	Baseline	Near peak: ~15Δ Hypo	Dist & Near: 1Δ–3Δ Hypo	a mild but functionally Significant Hypo
	Post-Intervention	Dist & Near: 3Δ–4Δ Hypo	Ortho	Dist: 1Δ Hypotropia Near: Orthophoria
DEM Reading Time (sec)	Baseline	H & V ~66 (H/V Ratio: 1.08)	H & V 60	H: 62 (100% errors), V: Omissions
	Post-Intervention	H & V: <60	H & V 50	H: 65, V: 60 (Accuracy Normalized)

Note: BCVA = Best-Corrected Visual Acuity; OD = Oculus Dexter (Right Eye); OS = Oculus Sinister (Left Eye); OU = Oculus Uterque (Both Eyes); cpm = cycles per minute; Eso = Esodeviation; Exo = Exodeviation; Hypo = Hypotropia; Ortho = Orthophoria; DEM = Developmental Eye Movement (H = Horizontal, V = Vertical). * Following a 6-week refractive adaptation period. ** Supported by a 10Δ base-out prism prescribed at Session 31.

## Data Availability

All data generated or analyzed during this study are included in this published article. Correspondence and requests for materials should be addressed to C.-Y.C.
